# The Diagnosis of Scarlet Fever

**Published:** 1907-05

**Authors:** A. R. Braunlich


### THE DIAGNOSIS OF SCARLET FEVER.

By A. R. BRAUNLICH, M.D.

The diagnosis of scarlet fever, although at times presenting many difficulties, can almost invariably be made positively if the case has been seen from the beginning and every symptom carefully noted.

In the following remarks, I shall confine myself entirely to deductions drawn from my own experience.

In arriving at a diagnosis, the course of events in a typical case should always be kept in mind, as there is a corresponding regularity in the appearance of the symptoms, whether the case be mild or severe. As a rule, the most severe cases of this disease live four or five days before death ensues. I have never seen a case die within twenty-four hours from onset. It might not be amiss to say here that scarlet fever practically never occurs during the first year of life. During the number of years that I have observed this disease,

I have seen but two cases under one year (both aged eleven months).

With the usual sudden onset, with disturbances in the stomach, nausea or vomiting, and occasionally convulsions, the diagnosis is based on the following objective symptoms: Condition of the tongue, the temperature, the pulse-rate, the rash and the condition of the throat.

The tongue, in the majority of cases during the entire course of this disease, shows nothing more than the ordinary fever coating. The real denuded strawberry tongue, i.e., a bright-red tongue with large papillæ and either no coating or only a slight coating on the posterior half, appears on the fourth or fifth day; and, occurring THEN, is of the greatest significance. Such a tongue seen on the first or second day of the disease should carry no weight as a diagnostic feature; for example, a case of pneumonia on the second day presented a diffuse general erythema, and a tongue typical of scarlet fever of about the fifth day. The rash being the only other suggestive symptom of scarlet fever, that disease was excluded. The desquamated tongue frequently persists for several weeks. The tongue, heavily coated, with red edges and prominent papillæ, which is so often said to be characteristic of the fever on the second and third days, occurs in so many other conditions that I consider it wholly unreliable as an aid to diagnosis.

Fever is, as a rule, present. There are exceptional cases where the temperature never goes above 99° F., the cases being typical in other respects. In young children the typical temperature curve is rarely seen. On the contrary, the temperature may be most irregular.

The pulse is invariably rapid, being elevated more than the usual eight or nine beats for each degree of fever. This rapidity of the pulse was present in the few nonfebrile cases that I have seen, and is often an aid to diagnosis.

The rash may appear within a few hours, and always appears within forty-eight hours after the onset of the disease, and regularly travels downward. It first appears on the neck and chest, at times not reaching the lower limbs until the second or third day.

If the case is seen as late as the third or fourth day, the rash on the trunk may have entirely faded. To me the vasomotor paresis of the capillary circulation has been of little value. The rash may be well developed on the face, and at times the circumoral pallor is present. I cannot say that my experience includes any cases of relapsing or recurring rashes. It must not be forgotten that the regular erythema may be accompanied by blotches resembling measles, and appearing usually on the extremities. With the erythema there is frequently a vesicular eruption, and when this is visible it may be well developed, especially on the extensor surfaces of the hands and feet. Of the irregular rashes frequently mentioned, I cannot say that I have ever seen one limited to one limb or to one side of the body which I was willing to regard as diagnostic of scarlet fever. There is, of course, no doubt that the erythema may be very faint on some parts of the body and of a deeper hue on other parts; and this condition may vary from day to day. In some severe cases, the rash is well developed only in the flexures of the joints. The cases of delayed appearance of the rash are usually cases of follicular tonsilitis or diphtheria occurring during the course of one of these diseases. In the majority of cases there is no similarity between the erythema of diphtheria and the rash of scarlet fever. In the majority of cases I have observed, a scarlatinal rash, with a rise in temperature, appearing in the course of diphtheria, has been due to a mixed infection. An immediate diagnosis, however, is not usually possible. Such cases sent to wards with scarlet fever cases have in no instance, to my knowledge, developed scarlet fever anew.

The "antitoxin rashes" occurring during convalescence of diphtheria, or several weeks after the injection of the serum, have raised notable difficulties of diagnosis. If such a rash be accompanied by a reinfection of the throat, it is probably due to infection with scarlet fever.

The throat always presents a different appearance from the normal of the suspected patient. In fact, it might be said that the disease begins with a sore throat, which may be so mild as not to be noted by the patient. On examination redness is seen on the tonsils and on the pharynx, and this latter progresses to the pillars and to the soft palate and at times to the hard palate. On the roof of the mouth, a punctate erythemia is frequently seen. Accompanying the redness, there is frequently an exudate on the tonsils, giving the appearance of a follicular tonsilitis or diphtheria.

It will be seen, from the foregoing, that the rash and the inflammation of the throat are necessary to make a diagnosis. In measles, malignant small-pox and varicella, erythema may precede the regular eruption by a few hours to several days. The antecedent erythema of malignant small-pox is always accompanied by subconjunctival hemorrhage, while that of measles is accompanied by the characteristic prodromal symptoms. The morbilliform eruption of rubella presents no difficulty for diagnosis, but the scarlatiniform rash of this disease frequently makes an exact diagnosis impossible. At present there are in this city a number of cases in which the diagnosis cannot now be made between German measles and scarlet fever. The patients with German measles apparently recover in about four days. They do not desquamate, or not freely, as in typical scarlet fever. One frequently is in doubt as to whether they are rubella, mild scarlet, or cases possibly of Filatow-Dukes' disease. (?) The only positive points of difference between these cases and plainly evident cases of scarlet fever is the speedy recovery and a pulse-rate lower than that found in scarlet of corresponding severity. Scarlatinal erythema with negative throats are probably not scarlet fever. Such rashes are seen with or without fever, and in a fever often do not appear at the proper period. They are sometimes seen in septic cases, following burns, again are drug-rashes from surgical applications, or from drugs taken internally, and again are the rashes of various infectious diseases, such as malaria, varicella, pneumonia, typhoid fever and small-pox. In measles and influenza, with naso-pharyngeal catarrh, the throat is red; but in both of these conditions, as well as in malaria, typhoid and pneumonia, the erythema is usually diffuse, not appearing like the typical scarlet fever rash, and the future course of the disease, with its accompanying symptoms, leads to a correct diagnosis.

The frequency of desquamation in many affections makes this condition in itself of little value. The time of its occurrence is of importance. Peeling in scarlet fever does not begin on the hands before the latter half of the second week. This time of desquamation, together with the history of relapses and recurrences, should be of assistance in exfoliating dermatitis, of which I have seen but very few cases. I am fully convinced that there are mild cases of scarlet fever where desquamation may be so scanty as to be entirely overlooked.—Archives of Pediatrics, March, 1907.

